# CALPHAD-Based Modelling of the Temperature–Composition–Structure Relationship during Physical Vapor Deposition of Mg-Ca Thin Films

**DOI:** 10.3390/ma16062417

**Published:** 2023-03-17

**Authors:** Philipp Keuter, Moritz to Baben, Shamsa Aliramaji, Jochen M. Schneider

**Affiliations:** 1 GTT-Technologies, Kaiserstraße 103, 52134 Herzogenrath, Germany; 2 Materials Chemistry, RWTH Aachen University, Kopernikusstr. 10, 52074 Aachen, Germanyschneider@mch.rwth-aachen.de (J.M.S.)

**Keywords:** CALPHAD, sublimation, PVD, sputtering, metals, magnesium, vapor pressure

## Abstract

The temperature-dependent composition and phase formation during the physical vapor deposition (PVD) of Mg-Ca thin films is modeled using a CALPHAD-based approach. Considering the Mg and Ca sublimation fluxes calculated based on the vapor pressure obtained by employing thermochemical equilibrium calculations, the experimentally observed synthesis-temperature trends in the thin-film composition and phase formation were reproduced. The model is a significant step towards understanding how synthesis parameters control composition and, therefore, phase formation in the PVD of metals with high vapor pressures.

## 1. Introduction

Physical vapor deposition (PVD) processes, such as magnetron sputtering, usually lead to non-equilibrium phase formation in as-grown thin films [1]. In recent years, however, it has been demonstrated that thin-film phase formation is closer to (at least para-) equilibrium than has often been assumed. Spencer demonstrated that robust thermodynamic descriptions of metastable phases can be used to predict phase formation in both metallic and nitride systems more than 20 years ago [2]. More recently, Chang et al. extended the underlying model assumptions through the numerical treatment of the surface diffusion kinetics relative to the deposition rate [3,4,5]. For reactive deposition, to Baben et al. showed that a para-equilibrium assumption, i.e., assuming the equal chemical potential of nitrogen in the gas phase and in the deposited thin film, can be used to understand the off-stoichiometry of as-deposited (Ti, Al)N_x_ thin films [6]. Here, we extend this further and consider the kinetics of metal sublimation using the example of magnetron-sputtered Mg-Ca thin films.

The sublimation of Mg during sputtering has been observed, for example, through the co-sputtering of Al-Mg-B thin films at substrate temperatures of 600 °C [7], limiting the temperature range for synthesis. The same effect was also observed for other metals exhibiting high vapor pressure at deposition temperature, such as Al [8], Mn [9], Sn [10], and Ge [10,11]. A pronounced variation in Mg-concentration depending on the synthesis temperature has also been reported for Mg-Ca thin films: minor variations in synthesis temperature yielded pronounced modifications in the thin films’ composition [12], revealing a complex synthesis–-composition–structure relationship when Mg is involved. Even though PVD is frequently applied to study the properties of elemental Mg [13,14] and Mg-based films [7,12,15,16,17,18,19,20,21,22], the sublimation behavior of Mg has only been considered qualitatively based on ground-state density-function-theory calculations [12]. In this work, thermochemical equilibrium calculations employing the CALPHAD (CALculation of PHAse Diagrams) approach are used to develop a predictive model of the temperature-dependent phase formation during Mg-Ca thin-film growth. In this model, sublimation is considered as the predominant desorption mechanism. The remaining differences obtained between the experimental results and the calculations within the temperature range for the phase-pure Mg_2_Ca formation are further investigated by correlative structural and chemical analyses of annealed combinatorial Mg-Ca thin films supporting a wider stoichiometry range of Mg_2_Ca than was expected from the CALPHAD model.

## 2. Materials and Methods

### 2.1. Theoretical Methods

**Figure 1:**
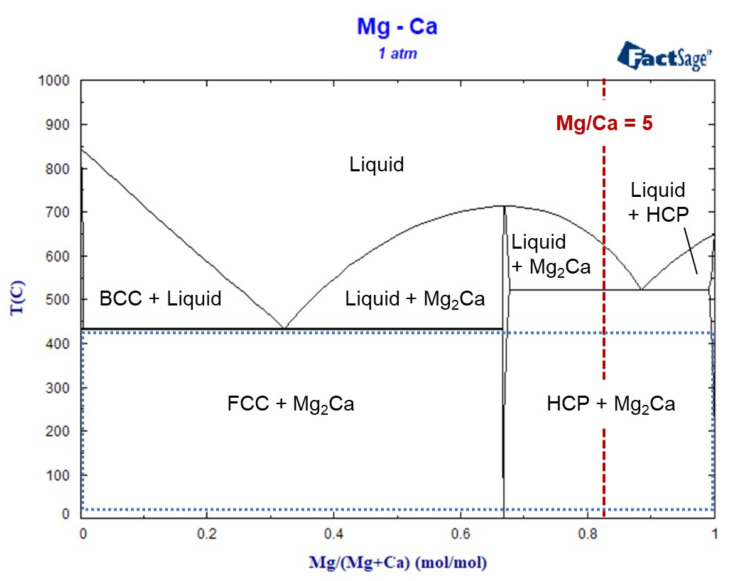
The Mg-Ca phase diagram indicating the input composition ratio of Mg/Ca (dashed red line) and studied temperature range (dotted blue line) in the model.

During magnetron sputtering, deposition and desorption occur concurrently. However, sublimation fluxes, constituting the desorption mechanism considered within this work, are affected by the chemical composition of the thin film, which is in turn affected by deposition and sublimation fluxes. To model this iteratively, consecutive steps of deposition and sublimation intervals of 10 s each were considered. Other interval lengths could also have been used, since this is primarily a numerical parameter. All calculations were performed considering a reference area of 1 m^2^. Thus, for each deposition step, 3.676 × 10^−4^ mol Mg and 7.352 × 10^−5^ mol Ca (Mg/Ca ratio of 5) are added to the thin-film composition, thus simulating the experimentally reported deposition flux using a composite Mg-Ca target exhibiting a Mg/Ca ratio of 5 [12]. Similar to the experiments [12], simulations were performed for temperatures between 20 °C and 420 °C. The Mg-Ca phase diagram calculated with FactSage [23] (version 8.1) using the FTlite database [24] with the highlighted input composition ratio and studied temperature range is shown in Figure 1.

The phase formation and vapor pressures of Mg and Ca, as the dominating gas species, were calculated based on thermochemical equilibrium calculations using the Equilib module of FactSage [23], version 8.1, with the commercial FTlite [24] database for the solid and liquid phases and FactPS [23] for the ideal gas phase. The thin-film composition and phase formation were updated after each deposition step, in which the individual thermochemical equilibrium calculations were performed using FactSage macros. By applying Hertz–Knudsen equation under the assumption of perfect vacuum [25,26]

(1) $${\dot{n}}_{i}=\frac{\alpha_{i}p_{i}N_{A}}{\sqrt{2\pi M_{i}RT}},$$

where $\alpha_{i}$ is the desorption coefficient, $p_{i}$ the equilibrium vapor pressure, $N_{A}$ Avogadro constant, $M_{i}$ the molar mass, R the ideal gas constant and T the temperature, the sublimation flux $\dot{n}$ and, consequently, the sublimated amount of the species i (Mg and Ca) from the solid film within 10 s was determined individually. For these calculations, the desorption coefficients $\alpha_{i}$ of Mg from hcp Mg and Ca from fcc Ca were each taken to be 1, while the Mg- and Ca-desorption coefficients from Mg_2_Ca were assumed to be 2/3 and 1/3, respectively, corresponding to their mole fractions. Due to the low solubilities of Mg in fcc Ca and Ca in hcp Mg, as displayed in Figure 1, these sublimation modes are not considered in the calculations. The remaining amounts of Mg and Ca after the sublimation step were subsequently used as inputs for the next deposition interval. Modeling of the phase formation was performed until convergence with a constant Mg/Ca ratio (fluctuations below 0.001) for 10 consecutive calculations was achieved. The final step was always a sublimation step.

### 2.2. Experimental Methods

To further investigate the stoichiometry range of the Mg_2_Ca (C14) Laves phase experimentally, combinatorial Mg-Ca thin films were synthesized in a high-vacuum laboratory-scale deposition chamber by direct-current magnetron sputtering using circular elemental Mg (99.95% purity) and Ca (99.5% purity) targets with a diameter of 50 mm at base pressures below 2 × 10^−5^ Pa. The Ca-target-cleaning procedure is described elsewhere [27]. Thin films were synthesized at room temperature (without intentional heating) at an Ar pressure of 0.4 Pa onto stationary Si (100) substrates, resulting in the formation of a composition gradient along the substrate. The deposition time was 10 min when applying constant Mg and Ca target powers of 200 W and 140 W, respectively. These films were subsequently annealed in the deposition chamber for 1 h to trigger crystallization at varying heater temperatures of up to 300 °C, denoted as annealing temperature. The corresponding substrate temperature, although not directly measured, was expected to be close to the set heater temperature, as discussed in the Results section of this paper.

Correlative chemical and structural analyses were performed by energy-dispersive X-ray spectroscopy (EDX) and X-ray diffraction (XRD) to study the composition-induced phase formation along the chemical gradient. The EDX was performed using an EDAX Genesis 2000 analyzer (EDAX Inc., Mahwah, NJ, USA) implemented in a JEOL JSM 6480 scanning-electron microscope (SEM, JEOL Ltd., Tokyo, Japan). The acceleration voltage was set to 10 kV and the measurement time was 120 s at a magnification of 1000×. For the structural analysis of the films, a Bruker D8 General Area Detection Diffraction System (GADDS, Bruker Corporation, Billerica, MA, USA) with Cu Kα radiation was employed. The voltage and current were set to 40 kV and 40 mA, respectively. The angle of incidence was fixed at 15°, whereas the 2θ range was measured from 15° to 75°. Peak positions based on the obtained diffractograms were determined by using the TOPAS software (version 3), employing a pseudo-Voigt II function. The lattice parameters *a* and *c* of the hexagonal C14 structure of Mg_2_Ca were calculated using the CellCalc software (version 2.10) considering the (110), (103), (112), (201), (110), (103), (201), and (004) reflections.

## 3. Results and Discussion

**Figure 2:**
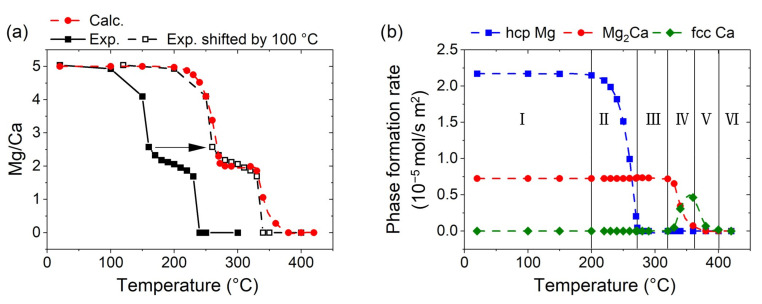
(**a**) Calculated temperature-dependent Mg/Ca ratio based on the applied model compared to the experimental data for deposited Mg-Ca thin films at varying temperatures (full black symbols), as reported in [12], and shifted by 100 °C (open black symbols). (**b**) Calculated phase-formation rate for hcp Mg, Mg_2_Ca, and fcc Ca as a function of the temperature separated into distinct regions with characteristic trends (I–VI).

To critically appraise the quality of the model, the calculated chemical composition expressed as the Mg/Ca ratio compared to the reported experimental results for the sputtered Mg-Ca thin films at varying substrate temperatures [12] is shown in Figure 2a.

There is a qualitative agreement in the overall trend, according to which both the experiment and the calculation indicated a two-step loss of Mg from the thin film, with a plateau spanning a temperature range of approximately 60 °C around a Mg/Ca ratio of 2. However, a pronounced deviation in the respective temperature ranges is obtained. Experimentally, a decrease in the Mg/Ca ratio was observed above 100 °C, while the onset of Mg loss is calculated to be above 200 °C. The discrepancy between the calculation and the experiments may have been caused by various factors. For instance, considering that the temperature calibration was conducted with a reference sample without plasma [12], which is expected to result in an additional heat impact on the substrate, it is expected that the actual substrate temperature during synthesis would be higher. Additionally, imperfect contact between the thermocouple and the substrate, resulting in an underestimation of the synthesis temperature, cannot be excluded, considering that the substrate temperature of 150 °C [12], for example, corresponded to a heater temperature of 260 °C. Therefore, it is noteworthy that increasing the experimental substrate temperature by 100 °C results in an excellent quantitative agreement between the experiment and the calculation. Hence, it is reasonable to assume that the experimental substrate’s temperature was, in fact, approximately 100 °C higher and, therefore, the temperatures used in the following text refer to the temperature used in the simulation or to the experimental calibration temperatures [12] shifted by +100 °C.

The composition-temperature trend, shown in Figure 2a, can be characterized by six distinct regions, which are discussed below, considering the predicted phase-formation rate as a function of the synthesis temperature, as shown in Figure 2b.

In region I, extending up to a temperature of 200 °C, both hcp Mg and intermetallic Mg_2_Ca forms at a temperature-independent rate, resulting in a film with an overall Mg/Ca ratio of 5 (see also Figure 2a). Between 200 and 270 °C (region II), the hcp Mg-phase-formation rate decreases rapidly with the increasing temperature, while the Mg_2_Ca formation rate remains constant. A further increase in temperature up to 320 °C (region III) yields the formation of phase-pure Mg_2_Ca at a temperature-independent rate. Region IV, ranging up to 360 °C, is characterized by a decreased phase-formation rate of Mg_2_Ca, while elemental fcc Ca begins to form. Between 360 and 380 °C (region V), the formation of Mg-containing phases ceases, but pure fcc Ca phase still formes at a reduced rate. At even higher temperatures (region VI), all the deposited species are sublimated and no film formes.

**Figure 3:**
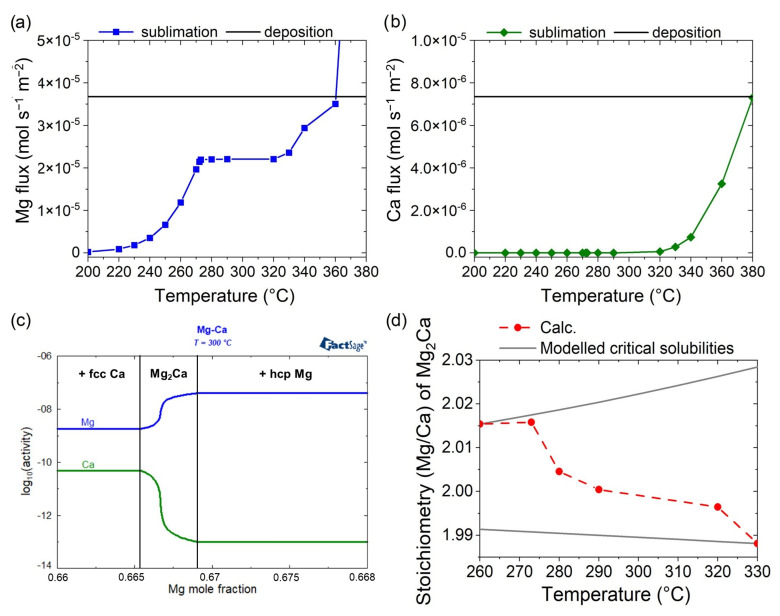
The calculated sublimation fluxes of Mg (**a**) and Ca (**b**) between 200 and 380 °C compared to the considered individual deposition fluxes in the model. (**c**) The Mg (blue) and Ca (green) vapor pressures (=activity) at 300 °C as a function of the Mg mole fraction and, thus, phase formation, as indicated by the vertical solid black lines separating regions of Mg_2_Ca + fcc Ca, pure Mg_2_Ca phase, and Mg_2_Ca + hcp Mg. (**d**) The calculated stoichiometry, as Mg/Ca ratio, of Mg_2_Ca focused on the temperature region with predicted pure Mg_2_Ca-phase formation (270–320 °C). The solid lines (gray) indicate the critical equilibrium solubilities, as modeled in the FTlite database [24] used for the thermochemical equilibrium calculations.

These temperature trends in composition and phase formation can be understood by considering the equilibrium vapor pressures of Mg and Ca (depicted for a constant temperature of 300 °C in Figure 3c, as an example) and the corresponding sublimation and deposition fluxes of Mg (Figure 3a) and Ca (Figure 3b), as calculated with the model.

The calculated sublimation fluxes in Figure 3a,b are affected by both the temperature and composition of the film, primarily through the changes in the equilibrium vapor pressures of Mg and Ca according to Equation (1). At a constant composition, the equilibrium vapor pressures generally increases with the increasing temperature, driving the increase in sublimation flux, as observed, for example, for Mg between 200 and 270 °C (see Figure 3a) and for Ca between 320 and 380 °C (Figure 3b) At a constant temperature, there is a constant vapor pressure of Mg for all the compositions in the two-phase region hcp Mg + Mg_2_Ca and Ca + Mg_2_Ca; the latter is significantly lower (see Figure 3c). In contrast, in the (temperature-dependent) composition range for the pure-Mg_2_Ca-phase formation, the vapor pressure of Mg decreases continuously and drastically when decreasing the Mg/Ca ratio (corresponding to a decreasing Mg mole fraction), as depicted in Figure 3c, using a temperature of 300 °C as an example, and a decrease in the Mg mole fraction from ~0.669 (corresponding to a Mg/Ca ratio of 2.02) to ~0.665 (corresponding to a Mg/Ca ratio of 1.99). Considering these observations, the emergence of the various temperature regions, as highlighted in Figure 2b, can be explained as follows. Up to a temperature of 200 °C (region I), no relevant sublimation is obtained due to the low vapor pressure of Mg at these temperatures (see near-zero Mg-sublimation flux at 200 °C in Figure 3a). Thus, in this temperature range, the ratio of the deposited Mg/Ca is conserved in the growing film. However, between 200 and 270 °C (region II), the deposited Mg is partly sublimated from the surface of the growing film, while the constant incoming deposition flux still exceeds the Mg solubility in the intermetallic Mg_2_Ca, so the hcp Mg forms as a second phase. Thus, in this range, the net film-forming flux (deposition flux–sublimation flux) of Mg is two times higher than that of the net film-forming flux of Ca (see Figure 3a,b). However, the previously discussed pronounced temperature dependency of the hcp Mg phase’s formation rate (see region II in Figure 2b) is driven by the exponential increase in the Mg-sublimation flux (Figure 3a). The temperature range, between 270 and 320 °C (region III in Figure 2b), in which the pure Mg_2_Ca phase forms, is characterized by the fact that the net film-forming flux of Mg becomes approximately two times that of the net film-forming flux of Ca, as shown in Figure 3c,d. Hence, all the Mg that cannot not be incorporated into the growing Mg_2_Ca film is sublimating from the surface. The obtained almost-constant Mg-sublimation flux between 270 and 320 °C, as shown in Figure 3a, is caused by the superimposition of the temperature-induced increase and the composition-induced decrease in the Mg vapor pressure (see Figure 3c), which balances each other out. The composition-induced decrease in the Mg vapor pressure is highlighted by the calculated Mg/Ca ratio of the phase-pure Mg_2_Ca as a function of the temperature (Figure 3d).

At 260 °C, the hcp Mg still forms as a second phase in addition to the intermetallic Mg_2_Ca phase with a maximum Mg solubility, as modeled in the FTlite database [24], while above this temperature, a continuous transition from Mg-rich Mg_2_Ca to Ca-rich Mg_2_Ca is observed upon rising temperature. The performed calculations demonstrate that, based on the thermochemical modeling in the FTlite database [24] as (Mg, Ca)_2_(Mg, Ca, vacancy), this transition is mainly driven by anti-site defects; this finding is in agreement with the defect-formation energies calculated using density functional theory [28]. Hence, the decrease in the Mg concentration in the phase-pure Mg_2_Ca yields a decrease in the Mg vapor pressure (Figure 3c) and, thus, compensated for the temperature-induced increase in the sublimation flux naturally occurring with the rising temperature. With a further increase in temperature above 320 °C, up to 360 °C (region IV), the vapor pressure of Mg above Mg_2_Ca and, thus, the Mg-sublimation flux (Figure 3a), rises continuously, resulting in the decreased phase-formation rate of Mg_2_Ca and in the formation of elemental fcc Ca (Figure 2b), since excess Ca, beyond the maximum Ca solubility in Mg_2_Ca, is now present at the growing thin-film surface. Despite the significant increase in the Ca-sublimation flux in this temperature range, enhancing the decrease in the Mg_2_Ca phase formation rate, the Mg-sublimation flux is still around 10 times higher than that of Ca at 360 °C (compare Figure 3a,b). Above 360 °C (region V), the Mg-sublimation flux finally exceeds the deposition flux, preventing the Mg incorporation into the growing film, causing the formation of phase-pure fcc Ca, despite the continuous Mg-deposition flux. The exponential increase in the Ca sublimation flux, however, leads to a near-zero phase-formation rate of Ca at 380 °C. At even higher temperatures (region VI), the net phase-formation fluxes of Mg and of Ca approache zero, inhibiting the formation of a thin film.

**Figure 4:**
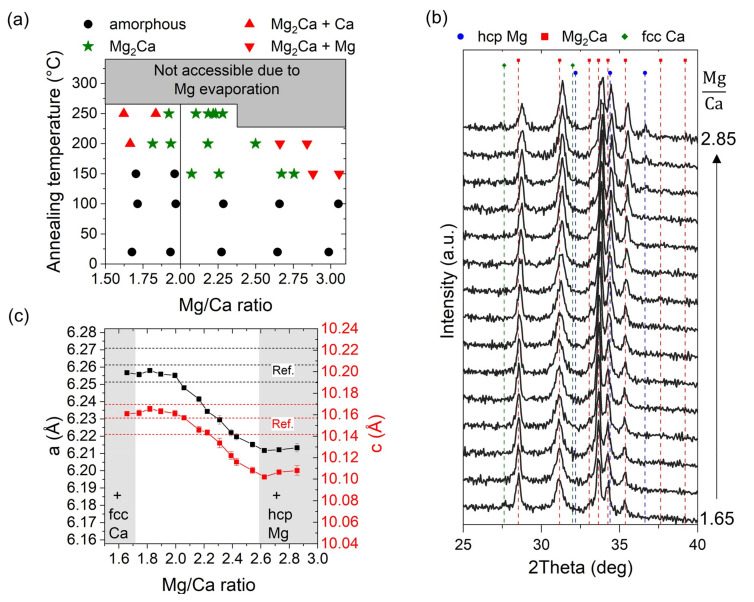
(**a**) Phase-formation map based on correlative chemical (EDX) and structural (XRD) analyses on compositionally graded Mg-Ca thin films as a function of annealing temperature and Mg/Ca ratio, indicating composition–temperature regions in which phase-pure Mg_2_Ca was obtained through XRD. (**b**) Diffractograms obtained by measuring along the Mg/Ca composition gradient starting from 1.65 (bottom) to 2.85 (top) of the thin film annealed at a heater temperature, of 200 °C. (**c**) Determined a and c lattice parameters of hexagonal (C14) Mg_2_Ca based on the diffractograms presented in (**b**). The color code on the different y-axes clearly explains what each line represents. For comparison, lattice-parameter values reported in the literature are added as dashed lines [29,32,33].

In contrast to the modeled stoichiometry range, ranging, for example, from 1.99 to 2.02 at 290 °C (see Figure 3d), the experimentally obtained composition range, in which phase-pure Mg_2_Ca was obtained through XRD, was reported to be between 1.7 to 2.2, based on the sublimation-dominated sputtering process of Mg-Ca thin films [12]. In general, controversial results regarding the stoichiometry range of Mg_2_Ca are reported in the literature, ranging from a line compound [29] to an off-stoichiometric formation [12,30,31]. To investigate the stoichiometry range of intermetallic Mg_2_Ca, compositionally graded Mg-Ca thin films were synthesized without intentional substrate heating and were subsequently annealed in vacuum without atmosphere exposure at varying temperatures. The results of the correlative composition–structure analyses along the chemical gradient of the samples are presented in Figure 4a.

The as-deposited combinatorial Mg-Ca film and the film after annealing at around 100 °C exhibited an X-ray amorphous structure spanning the whole Mg/Ca composition gradient, ranging from around 1.6 to 3. Upon annealing at around 150 °C for 1 h, the formation of Mg_2_Ca and Mg crystallites was observed with a Mg/Ca ratio > 2.0, while the film with a Mg/Ca ratio < 2.0 remained amorphous, potentially due to kinetically limited crystallization. A further increase in the annealing temperature to 200 °C yielded a crystalline film along the entire chemical gradient present on the 2″ wafer, still covering a Mg/Ca range from around 1.6 to 2.9, suggesting that sublimation does not play a major role in this temperature range. After an annealing process at a heater temperature of 250 °C, however, the composition gradient decreased to a Mg/Ca ratio of 1.6 to 2.3, indicating sublimation on the Mg-rich side of the deposited wafer. The temperatures discussed here, indicating the transition from no substantial Mg sublimation to significant Mg loss, are in good agreement with onset, modeled in this study, of Mg sublimation above 200 °C during the sputtering of the Mg-Ca thin films using intentional heating during deposition (Figure 2). At even higher annealing temperatures, no Mg/Ca ratio within the range presented here was obtained; this can be attributed to severe Mg sublimation. The fully crystalline compositionally graded Mg-Ca thin film without signs of pronounced sublimation (indicated annealing temperature of 200 °C) was consequently analyzed in finer composition steps by XRD, as presented in Figure 4b. For a Mg/Ca ratio of 1.65, all the main peaks can be assigned to Mg_2_Ca, while the presence of fcc Ca as a second phase is indicated by the peak at ~27.6° (JCPDS 23-0430). Upon a further increase in the Mg/Ca ratio, the fcc Ca peak vanished and the formation of phase-pure Mg_2_Ca was obtained through XRD. It needs to be considered, however, that due to the high reactivity of the fcc Ca with the atmosphere (an exposure that was required for the XRD analysis), the formation of nanocrystalline/amorphous Ca(OH)_2_ is expected [12,27]. Hence, no attempt is conducted here to determine a composition limit for the formation of the pure Mg_2_Ca phase on the Ca-rich side. When increasing the Mg/Ca ratio, however, a continuous peak shift to larger diffraction angles was observed, indicating lattice-parameter variations for hexagonal (C14) Mg_2_Ca, which is considered a reliable indicator of the off-stoichiometric formation of the phase. For even larger Mg concentrations, hcp Mg formed as a second phase, as indicated by the emergence of a characteristic peak at 36.6° (JCPDS 35-0821). Based on the diffraction data, the Mg_2_Ca lattice parameters *a* and *c*, as presented in Figure 4c, were determined.

The almost constant lattice parameters, *a* and *c*, obtained for a Mg/Ca ratio between 1.65 and 2.0 (Mg/Ca), lie within the reported lattice-parameter ranges, which vary from 6.2514 to 6.2709 Å and from 10.1417 to 10.1696 Å, respectively [29,32,33]. For a Mg/Ca ratio between 2.0 and 2.5, a continuous decrease in lattice parameters *a* and *c* was obtained, while a further increase in the Mg concentration yielded the formation of hcp Mg as a second phase, based on the XRD analysis. The decrease in the lattice parameters with the increasing Mg concentration in the Mg_2_Ca is in line with the atomic-size ratio of 1.231 (Ca/Mg) [34], according to which a decrease in lattice parameters is expected upon Mg anti-site defect formation (Mg atoms on a Ca site). Considering the accuracy of standardless EDX quantification, as applied here, of ±2% [35], this paper does not aim to provide the precise phase-formation range of Mg_2_Ca. Nevertheless, the results indicate an extensive (at least metastable [2,3]) phase-formation range, in line with that reported by Suzuki et al., who observed a solubility of excess Mg of around 4.5 at.% [31] (corresponding to a Mg/Ca ratio of 2.47), which is significantly wider than that modeled in the FTlite database [24]. However, it was not ensured that the thin film was sufficiently equilibrated after annealing 1 h at 200 °C. Consequently, the observed difference between the calculated and modeled results in the temperature region of phase-pure Mg_2_Ca formation is at least partly attributable to the limited stoichiometry range in the model, which did not account for the extended, potentially metastable, solubility obtained experimentally.

## 4. Conclusions

By employing CALPHAD-based thermochemical equilibrium calculations and the Hertz–Knudsen equation to describe the sublimation, the composition and the phase formation during the sputtering of Mg-Ca thin films were modeled as a function of the synthesis temperature and the Mg/Ca ratio. The agreement between the model calculations and the experimental results reported in the literature suggests that temperature-induced sublimation from the surface of the growing film is the predominant mechanism determining the composition–structure relationship during sputtering in this system. Thus, it was demonstrated that the film composition and structure can be predicted in sublimation-dominated-deposition scenarios by applying thermochemical equilibrium calculations. By explicitly considering the synthesis temperature and the deposition fluxes of the individual species in the model, it is expected that the model can serve for the prediction of suitable synthesis parameters for the sputtering of material systems, which are prone to sublimation. This study corroborates the results of previous studies concerning the applicability of (para-) equilibrium calculations in PVD [2,3,4,5,6] and is, thus, a significant step towards understanding how synthesis parameters control composition and, therefore, phase formation in PVD.

## Data Availability

Data available on request from the corresponding author.

## References

[1] Löffler F.H. (1992). Formation of Non-Equilibrium Phases in the PVD Process. Vacuum.

[2] Spencer P. (2001). Thermodynamic Prediction of Metastable Coating Structures in PVD Processes. Int. J. Mater. Res..

[3] Chang K., Music D., to Baben M., Lange D., Bolvardi H., Schneider J.M. (2016). Modeling of Metastable Phase Formation Diagrams for Sputtered Thin Films. Sci. Technol. Adv. Mater..

[4] Chang K., to Baben M., Music D., Lange D., Bolvardi H., Schneider J.M. (2015). Estimation of the Activation Energy for Surface Diffusion during Metastable Phase Formation. Acta Mater..

[5] Liu S., Chang K., Mráz S., Chen X., Hans M., Music D., Primetzhofer D., Schneider J.M. (2019). Modeling of Metastable Phase Formation for Sputtered Ti1-XAlxN Thin Films. Acta Mater..

[6] to Baben M., Hans M., Primetzhofer D., Evertz S., Ruess H., Schneider J.M. (2017). Unprecedented Thermal Stability of Inherently Metastable Titanium Aluminum Nitride by Point Defect Engineering. Mater. Res. Lett..

[7] Wu Z., Bai Y., Qu W., Wu A., Zhang D., Zhao J., Jiang X. (2010). Al–Mg–B Thin Films Prepared by Magnetron Sputtering. Vacuum.

[8] Beckers M., Höglund C., Baehtz C., Martins R.M.S., Persson P.O.Å., Hultman L., Möller W. (2009). The Influence of Substrate Temperature and Al Mobility on the Microstructural Evolution of Magnetron Sputtered Ternary Ti–Al–N Thin Films. J. Appl. Phys..

[9] Herrig F. (2019). Ab Initio Guided Design of Thin Film Model Systems for FeMn Based Steels. Ph.D. Thesis.

[10] Högberg H., Emmerlich J., Eklund P., Wilhelmsson O., Palmquist J.P., Jansson U., Hultman L. (2006). Growth and Property Characterization of Epitaxial MAX-Phase Thin Films from the Tin+1(Si, Ge, Sn)Cn Systems. Adv. Sci. Technol..

[11] Wilhelmsson O., Eklund P., Högberg H., Hultman L., Jansson U. (2008). Structural, Electrical and Mechanical Characterization of Magnetron-Sputtered V–Ge–C Thin Films. Acta Mater..

[12] Keuter P., Karimi Aghda S., Music D., Kümmerl P., Schneider J.M. (2019). Synthesis of Intermetallic (Mg1−x, Alx)2Ca by Combinatorial Sputtering. Materials.

[13] Ham B., Junkew A., Bufford D., Arróyave R., Zhang X. (2014). Fabrication of Porous and Pillar-Shaped Mg by Magnetron Sputtering. Thin Solid Film..

[14] Structure and Corrosion of Magnetron Sputtered Pure Mg Films on Silicon Substrates—Störmer. 2007. Plasma Processes and Polymers. Wiley Online Library. https://onlinelibrary.wiley.com/doi/10.1002/ppap.200731405.

[15] Grigucevičienė A., Leinartas K., Juškėnas R., Juzeliūnas E. (2005). Structure and Initial Corrosion Resistance of Sputter Deposited Nanocrystalline Mg–Al–Zr Alloys. Mater. Sci. Eng. A.

[16] Bouaziz O., Billard A. (2007). Structure–Mechanical Properties Relationships of Co-Sputter Deposited Iron–Magnesium Coatings. Surf. Coat. Technol..

[17] Ludwig A., Cao J., Dam B., Gremaud R. (2007). Opto-Mechanical Characterization of Hydrogen Storage Properties of Mg–Ni Thin Film Composition Spreads. Appl. Surf. Sci..

[18] Schmuelling G., Winter M., Placke T. (2015). Investigating the Mg–Si Binary System via Combinatorial Sputter Deposition as High Energy Density Anodes for Lithium-Ion Batteries. ACS Appl. Mater. Interfaces.

[19] Olk C.H., Haddad D.B. (2007). Growth and Structure of a Combinatorial Array of Mixed-Phase Magnesium–Aluminum Thin-Film Alloys. Appl. Phys. A.

[20] Shedden B.A., Samandi M., Window B. (1997). Stoichiometry of Unbalanced Magnetron Sputtered Al–Mg Alloy Coatings. Surf. Coat. Technol..

[21] Yamada Y., Bao S., Tajima K., Okada M., Yoshimura K. (2009). Optical Properties of Switchable Mirrors Based on Magnesium-Calcium Alloy Thin Films. Appl. Phys. Lett..

[22] Hans M., Keuter P., Saksena A., Sälker J.A., Momma M., Springer H., Nowak J., Zander D., Primetzhofer D., Schneider J.M. (2021). Opportunities of Combinatorial Thin Film Materials Design for the Sustainable Development of Magnesium-Based Alloys. Sci. Rep..

[23] Bale C.W., Chartrand P., Degterov S.A., Eriksson G., Hack K., Ben Mahfoud R., Melançon J., Pelton A.D., Petersen S. (2002). FactSage Thermochemical Software and Databases. Calphad.

[24] Jung I.-H., Zhu Z., Kim J., Wang J., Chartrand P., Pelton A. (2017). Recent Progress on the Factsage Thermodynamic Database for New Mg Alloy Development. JOM.

[25] Safarian J., Engh T.A. (2013). Vacuum Evaporation of Pure Metals. Metall. Mater. Trans. A.

[26] Hołyst R., Litniewski M., Jakubczyk D. (2015). A Molecular Dynamics Test of the Hertz–Knudsen Equation for Evaporating Liquids. Soft Matter.

[27] Aliramaji S., Keuter P., Neuß D., Hans M., Primetzhofer D., Depla D., Schneider J.M. (2023). Effect of Growth Temperature and Atmosphere Exposure Time on Impurity Incorporation in Sputtered Mg, Al, and Ca Thin Films. Materials.

[28] Shao L., Shi T.-T., Zheng J., Pan X.-Z., Tang B.-Y. (2015). The Native Point Defects in C14 Mg_2_Ca Laves Phase: A First-Principles Study. Intermetallics.

[29] Kevorkov D., Medraj M., Li J., Essadiqi E., Chartrand P. (2010). The 400 °C Isothermal Section of the Mg–Al–Ca System. Intermetallics.

[30] Freund M., Andre D., Zehnder C., Rempel H., Gerber D., Zubair M., Sandlöbes-Haut S., Gibson J.S.K.-L., Korte-Kerzel S. (2021). Plastic Deformation of the CaMg_2_ C14-Laves Phase from 50–250 °C. Materialia.

[31] Suzuki A., Saddock N.D., Jones J.W., Pollock T.M. (2006). Phase Equilibria in the Mg-Al-Ca Ternary System at 773 and 673 K. Metall. Mater. Trans. A.

[32] Amerioun S., Simak S.I., Häussermann U. (2003). Laves-Phase Structural Changes in the System CaAl_2_-XMgx. Inorg. Chem..

[33] Kozlov A., Ohno M., Arroyave R., Liu Z.K., Schmid-Fetzer R. (2008). Phase Equilibria, Thermodynamics and Solidification Microstructures of Mg–Sn–Ca Alloys, Part 1: Experimental Investigation and Thermodynamic Modeling of the Ternary Mg–Sn–Ca System. Intermetallics.

[34] Xie Z., Chauraud D., Bitzek E., Korte-Kerzel S., Guénolé J. (2021). Laves Phase Crystal Analysis (LaCA): Atomistic Identification of Lattice Defects in C14 and C15 Topologically Close-Packed Phases. J. Mater. Res..

[35] Pinard P.T., Protheroe A., Holland J., Burgess S., Statham P.J. (2020). Development and Validation of Standardless and Standards-Based X-ray Microanalysis. IOP Conf. Ser. Mater. Sci. Eng..

